# Rapid development of mature vocal patterns of ultrasonic calls in a fast-growing rodent, the yellow steppe lemming (*Eolagurus luteus*)

**DOI:** 10.1371/journal.pone.0228892

**Published:** 2020-02-11

**Authors:** Daria D. Yurlova, Ilya A. Volodin, Olga G. Ilchenko, Elena V. Volodina

**Affiliations:** 1 Department of Vertebrate Zoology, Faculty of Biology, Lomonosov Moscow State University, Moscow, Russia; 2 Scientific Research Department, Moscow Zoo, Moscow, Russia; University of Sussex, UNITED KINGDOM

## Abstract

Ultrasonic vocalizations (USV) of laboratory rodents may serve as age-dependent indicators of emotional arousal and anxiety. Fast-growing Arvicolinae rodent species might be advantageous wild-type animal models for behavioural and medical research related to USV ontogeny. For the yellow steppe lemming *Eolagurus luteus*, only audible calls of adults were previously described. This study provides categorization and spectrographic analyses of 1176 USV calls emitted by 120 individual yellow steppe lemmings at 12 age classes, from birth to breeding adults over 90 days (d) of age, 10 individuals per age class, up to 10 USV calls per individual. The USV calls emerged since 1^st^ day of pup life and occurred at all 12 age classes and in both sexes. The unified 2-min isolation procedure on an unfamiliar territory was equally applicable for inducing USV calls at all age classes. Rapid physical growth (1 g body weight gain per day from birth to 40 d of age) and the early (9–12 d) eyes opening correlated with the early (9–12 d) emergence of mature vocal patterns of USV calls. The mature vocal patterns included a prominent shift in percentages of chevron and upward contours of fundamental frequency (f0) and the changes in the acoustic variables of USV calls. Call duration was the longest at 1–4 d, significantly shorter at 9–12 d and did not between 9-12-d and older age classes. The maximum fundamental frequency (f0max) decreased with increase of age class, from about 50 kHz in neonates to about 40 kHz in adults. These ontogenetic pathways of USV duration and f0max (towards shorter and lower-frequency USV calls) were reminiscent of those in laboratory mice *Mus musculus*.

## Introduction

Ultrasonic vocalizations (USV) of laboratory rodents represent age-dependent indicators of animal emotional arousal [1–6] and may serve for modeling human diseases and the evaluation of drugs and medicaments effects [7–20]. The overwhelmingly preferred mice USV model [1,8,10,21–24] is applicable for USV ontogeny [7,16,19,20,25–33]. However, in spite of the numerous wild-type and mutant strains of laboratory mice *Mus musculus* [18,26,27,34–37], the mice model does not suffice for all spectrum of biomedical research based on analyses of USV [3,5,38,39]. For example, the mice model does not provide distinctive vocal correlates of negative and positive emotions [5], which are embedded in rat model of 22 kHz and 50-kHz USV calls [3].

In addition, animal strains selected for behaviour demonstrate destabilization effects after breeding in a series of generations. Destabilization effects result in unexpected changes of behaviour during experiments, as e.g. in catatonic rats *Rattus norvegicus* [40] and changes in morphological traits, as e.g. in silver fox *Vulpes vulpes* selected for tame behaviour [41,42] or in rats selected for call rate [43]. In silver fox, selection for tame behaviour also affects vocalization [44,45]. At the same time, in some rodent species, as e.g. golden hamsters *Mesocricetus auratus*, vocal traits are relatively resistant to the destabilizing effects across generations [46]. These facts explain a growing research interest to non-traditional wild-type animal models of vocal ontogeny [1,12,39,46–53].

The ontogenetic changes of USV calls may differ between species [12,39,49,54–56]. The age-related growth affects USV acoustic variables [12,26,29,39,49,55–57], percentage of different contour shapes (flat, chevron, wave, upward, downward) [7,39,49,54,58–65] and percentage of different nonlinear phenomena: frequency jumps, biphonations and subharmonics [39,49].

Most frequent nonlinear phenomena in rodent USV calls are frequency jumps, recognizable by breaks of a continuous USV contour to two or more notes [7,39,49,54,66]. Biphonations (recognizable by two independent fundamental frequencies and their combinatory bands) and subharmonics (recognizable by presence of frequency bands of ½ of f0) are rare in rodent USV calls [39,67].

Key acoustic variables, fundamental frequency (f0) and duration, reflecting developmental changes of USV calls, display different ontogenetic trajectories across species. For example, in ontogeny of laboratory rat, duration overall increases while the f0 decreases [12,55,56]. In ontogeny of laboratory mouse, both duration and f0 decrease [26,29]. In ontogeny of fat-tailed gerbil *Pachyuromys duprasi*, duration decreases whereas f0 increases [39]. So far, there are no explaining hypotheses for the differences in the ontogenetic trajectories of the key acoustic variables between taxa.

In some rodent species, both pups and adults may produce USV calls when isolated from conspecifics [5,37,49,68–71]. Species producing isolation-induced USV calls in the same context of short-term isolation at unfamiliar territory across age classes, might be especially good candidates for the ontogenetic studies.

The yellow steppe lemming *Eolagurus luteus* is a diurnal medium-sized Arvicolinae rodent species [72,73] inhabiting steppe regions of Mongolia, North-Eastern China, Eastern Kazakhstan and Southern Altai (Russia) [74–77]. Yellow steppe lemming laboratory populations are maintained in scientific institutions and zoos [78–80].

For a natural population of yellow steppe lemmings in the East Kazakhstan, the average reported body length was about 147 mm in both sexes; male body weight ranged of 75–124 g (100 g on average) and female body weight of 60–139 (about 102 g). Both males and females were fertile since 40 d of age, gestation period comprised 17–18 d, litter size varies from 3 to 5 pups [76].

Similarly, in a laboratory population originated from the East Kazakhstan (1–4 generations in captivity), adult female weighted on average 80 g and displayed a high breeding rate: age of first conception at 34 d, gestation period 17–18 d, the average inter-birth interval 29 days, and litter size 3–10 pups; males were fertile since 38 d of age [78]. Pups displayed fast growth: body weight gain from 3.9 g at birth to 27.4 g post-weaning (at 13–20 d of age) in either sex and early eyes opening (at 12 d of age) [78].

Adult and subadult yellow steppe lemmings produce audible quiet and sharp squeaks of 1.5–4.0 kHz fundamental frequency during the experimental “different sex interaction” procedure [80]. Compared to adults, squeaks of subadults are shorter (0.052 s *vs* 0.076 s) and higher-frequency (2.3 kHz *vs* 1.7 kHz) [80]. In addition to the audible acoustic communication, this species was previously investigated for their potential to visual communication: peculiar retina histology compared to other diurnal rodent species, the Brand’s vole *Lasiopodomys brandtii* and the great gerbil *Rhombomys opimus* [81].

Ultrasonic vocalization of yellow steppe lemmings was not investigated so far. The aims of this study were 1) to categorize isolation-induced USV calls of yellow steppe lemmings; 2) to describe any vocal features unique to each of 12 age classes from birth to mature adults; 3) to determine the developmental time point at which mature vocal pattern emerge; 4) to estimate relationships between body size, age, sex and the USV acoustics. This is the first description of USV calls and of vocal ontogeny in yellow steppe lemming, and the first comprehensive catalogues of vocal development of the isolation-related USV calls from birth through maturity for any Arvicolinae rodent.

## Material and methods

### Ethics statement

This study was part of the research program of the Scientific Research Department of Moscow Zoo. The three authors are zoo staff members, so no special permission was required for them to work with animals in Moscow Zoo. All study animals belonged to the laboratory collection of Moscow Zoo. The experimental procedure has been approved by the Committee of Bio-ethics of Lomonosov Moscow State University, research protocol # 2011–36. We adhered to the ‘Guidelines for the treatment of animals in behavioural research and teaching’ [82] and to the laws on animal welfare for scientific research of the Russian Federation, where the study was conducted. For handling of animals during measurements, we adhered to the guidelines ‘Hand restraint of wildlife’ [83]. No one single animal suffered due to data collection.

### Study site and subjects

The USV calls were collected from 120 members of a captive population of yellow steppe lemmings at Moscow Zoo, Moscow, Russia, in February-July 2018. All subjects were descendants of 7 individuals, obtained by Moscow Zoo in autumn 2016—spring 2017 from a natural population in East Kazakhstan (48^о^10’N, 84^о^25’E).

Before parturition, females of the captive population were checked three times per week for the appearance of a litter, and birth dates as well as the number of pups were recorded. The day of birth was considered zero day of pup life. The subjects comprised 110 pups from 52 litters between 1 and 60 days and 10 adults (5 males, 5 females) older 90 days with breeding experience. Study pups were offspring of 10 breeding pairs of 1–2 generation in captivity from 1 to 8 litters per pair, 5.2 ± 2.6 litters per pair on average. Study pups were sexed after 20–25 days of age, based on visible testicles in males or vagina in females. All 10 study adults were parents of study pups and were members of 7 breeding pairs. Subjects belonged to 12 age classes: 1–4 d, 5–8 d, 9–12 d, 13–16 d, 17–20 d, 21–24 d, 28–32 d, 33–36 d, 37–40 d; 41–60 d and over 90 d (adults), 10 individuals per age class from 5–7 (5.4 ± 0.7 on average) litters per age class, from 1 to 3 (1.86 ± 0.66) pups per litter.

We did not use the longitudinal approach with the same individuals repeatedly tested in each age class, because preliminary observations of zoo staff suggested that regular taking the same pups of yellow steppe lemming for weighing resulted in growth retardation of pups from the experimental litters compared to the pups which were not taken for weighing. So, we selected to use the cross-sectional approach with many non-overlapping age classes for avoiding the effects of the repeated testing on development of the experimental pups.

### Animal housing

The subject animals were kept under a natural light regime at room temperature (22–25°C), in family groups consisting of two parents and littermates of 1–3 subsequent litters. Pups at the age until 20–30 d, used in the experiments, were kept in family groups with their parents. The older pups (from 20 to 60 d) were kept with their parents, sometimes in a group could present pups of the next younger litter. At 30–60 d of age, the adolescents were separated from the parents; the separated adolescents did not participated in experiments. The experimental adults were always breeding parents of family groups. The breeding adults were individually chip-marked, whereas the small size of pups also prevented individual chip marking for ethical reasons until 20–25 d of age.

The animals were housed in wire-and-glass cages of 50х100х35 cm, with a bedding of sawdust of 8–10 cm and hay and various wooden shelters and cardboard pipes of 4-5-cm diameter as enrichment. They received custom-made small desert rodent chow with mineral supplements and fruits and vegetables *ad libitum* as a source of water.

### Experimental procedure and USV recording

All acoustic recordings were conducted in a separate room where no other animals were present, at room temperature 22–25°C during daytime, at the same level of background noise. For USV recordings (sampling rate 384 kHz, 16 bit resolution) we used a Pettersson D1000X recorder with built-in microphone (Pettersson Electronik AB, Uppsala, Sweden). The microphone was established stationary at distance 35 cm above the animal. The obtained recordings had a high signal/noise ratio, the reverberation practically lacked. Recording of each trial was stored as a wav-file.

Each subject animal participated only in one experimental trial. Each individual was tested singly. Immediately before an experimental trial, the focal animal was taken from the home cage and transferred in a small clean plastic hutch to the experimental room within the same floor of the building. Time from removal of the focal animal from the cage to the start of an experimental trial did not exceed 60 s. During the trial, the animal just was isolated in an experimental setup, either clean plastic hutch (190x130x70 mm for 1–12 d pups) or in a plastic cylinder without bottom (diameter 193 mm, high 170 mm for 13–60 d pups and adults), standing on even plastic table surface. Both the plastic huge and cylinder were open from above, i.e. from the side where the microphone was placed. The recording started, when the focal animal was placed to the experimental setup and lasted 120 s. Aside isolation, the focal animals experienced also a cooling, due to the imperfect thermoregulation of 1–12 d pups with still poorly developed fur cover. No additional actions from the experimenter were applied toward the animal, the animals could move freely.

After the trial, the focal animal was weighed and measured for body length, head length, foot length and tail length. For weighing, we used G&G TS-100 electronic scales (G&G GmbH, Neuss, Germany), accurate to 0.01 g. Weighing was done in the same plastic hutch which was used for transferring the animal to the experimental setup. For the lengths measurements, we used electronic calipers (Kraf Tool Co., Lenexa, Kansas, US), accurate to 0.01 mm. We measured body length of the hand-held animal from the tip of the snout to the anus, and head length from the tip of the snout to the occiput. We measured foot length from the heel to the tip of the middle toe, and tail length from anus to the tip to the tail. These measurements were repeated three times and the mean value was taken for analysis. The body variables and body weight were measured as proxies of body size for further comparison with the USV acoustic variables.

If more than one littermate per litter was tested, after the end of a trial, the focal pup was placed to a heating hutch with a bedding of a cotton fabric, standing in the neighboring room. Experimental trials with all focal littermates were done consequently in the same manner. Then all of them were simultaneously returned to their home cage to their parents; the time of pup stay out of the nest did not exceed 30 min. The adults were taken from their home cages before experiments with a clean plastic glass and returned to the cage after the test trial. The experimental setup was rubbed with napkin wetted with alcohol after each experimental trial, to avoid effect of smell on USV of the next focal animal in the next experimental trial [84–86].

### USV call samples

We use the term “isolation call” to refer to any USV call produced by individual subject yellow steppe lemming of any age class during the experimental 2-min isolation procedure. Using visual inspection of spectrograms of acoustic files created with Avisoft SASLab Pro software (Avisoft Bioacoustics, Berlin, Germany) we selected 10 USV calls per individual, however three pups provided only 9, 3, 3 USV calls, and one adult provided only 1 USV call. We took calls randomly among those considered eligible, of high sound-to-noise ratio and without superimposed noise from different parts of each 120 s recording, avoiding taking calls following each other. Call frequency contour and presence of nonlinear phenomena were not considered as selection criteria. In total, for the 120 study animals of all the 12 age classes, we selected for acoustic analyses 1176 USV calls.

### Acoustic analysis

Measurements of acoustic variables of pup and adult USV calls have been conducted with Avisoft and exported to Microsoft Excel (Microsoft Corp., Redmond, WA, USA). As minimum fundamental frequency of USV calls always exceeded 10 kHz, before measurements all wav-files were subjected to 10 kHz high-pass filtering, to remove low-frequency noise.

**Fig 1:**
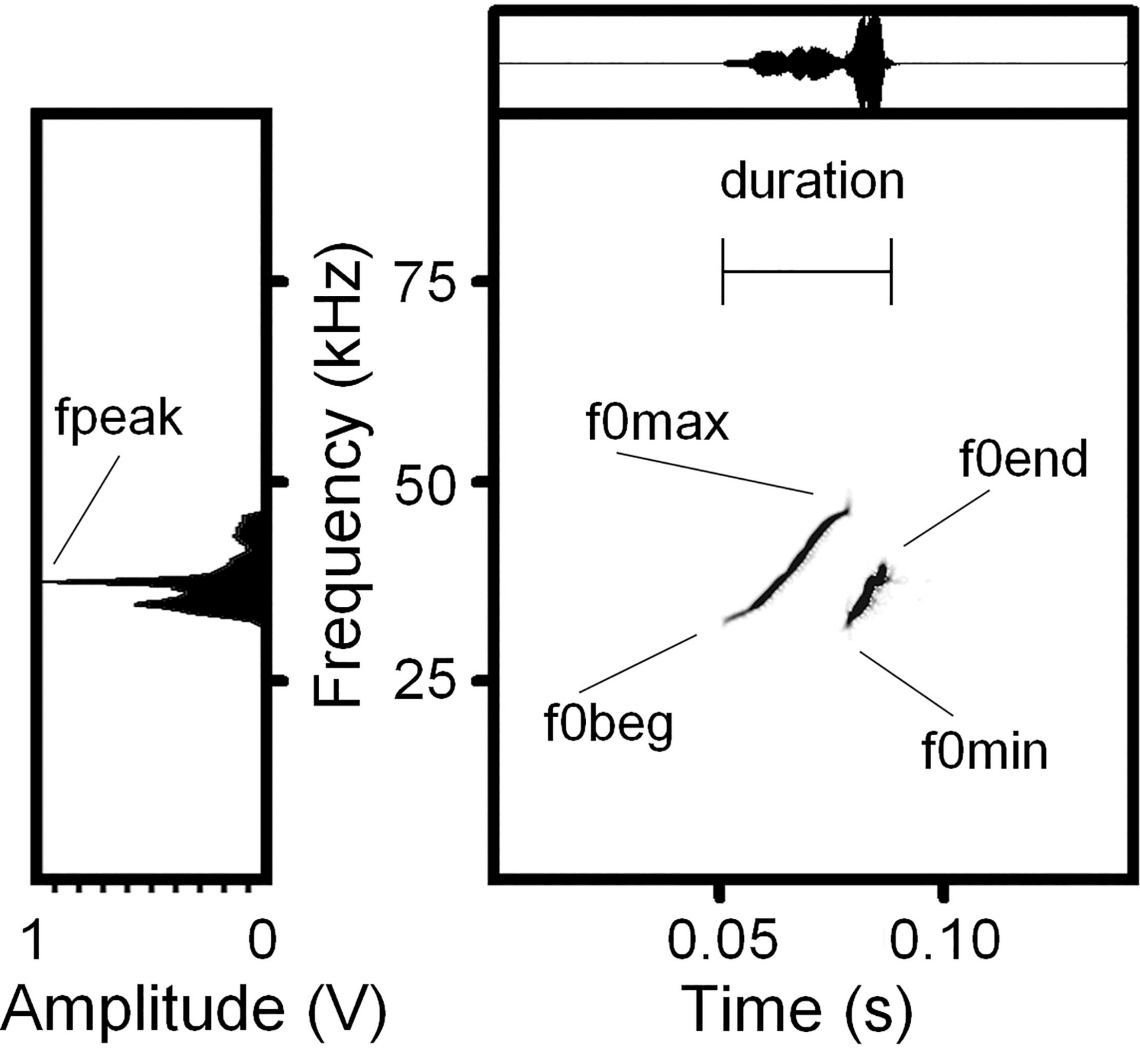
Measured variables for yellow steppe lemmings USV calls exemplified by a pup USV call with frequency jump and upward contour. Spectrogram (right) and mean power spectrum of the entire call (left). Designations: duration–call duration; f0beg–the fundamental frequency at the onset of a call; f0end–the fundamental frequency at the end of a call; f0max–the maximum fundamental frequency; f0min–the minimum fundamental frequency; fpeak–the frequency of maximum amplitude. Spectrogram was created using sampling frequency 192 kHz, Hamming window, FFT 1024 points, frame 50% and overlap 93.75%.

For each USV call, we measured, in the spectrogram window of Avisoft (sampling frequency 384 kHz, Hamming window, FFT 1024 points, frame 50%, overlap 87.5%, providing frequency resolution 375 Hz and time resolution 0.33 ms), the duration with the standard marker cursor, and the maximum fundamental frequency (f0max), the minimum fundamental frequency (f0min), the fundamental frequency at the onset of a call (f0beg), and the fundamental frequency at the end of a call (f0end) with the reticule cursor (Fig 1 and S1 Table). For each USV call, we measured, in the power spectrum window of Avisoft, the frequency of maximum amplitude (fpeak) from the call’s mean power spectrum (Fig 1 and S1 Table).

### USV contour shapes and nonlinear vocal phenomena

**Fig 2:**
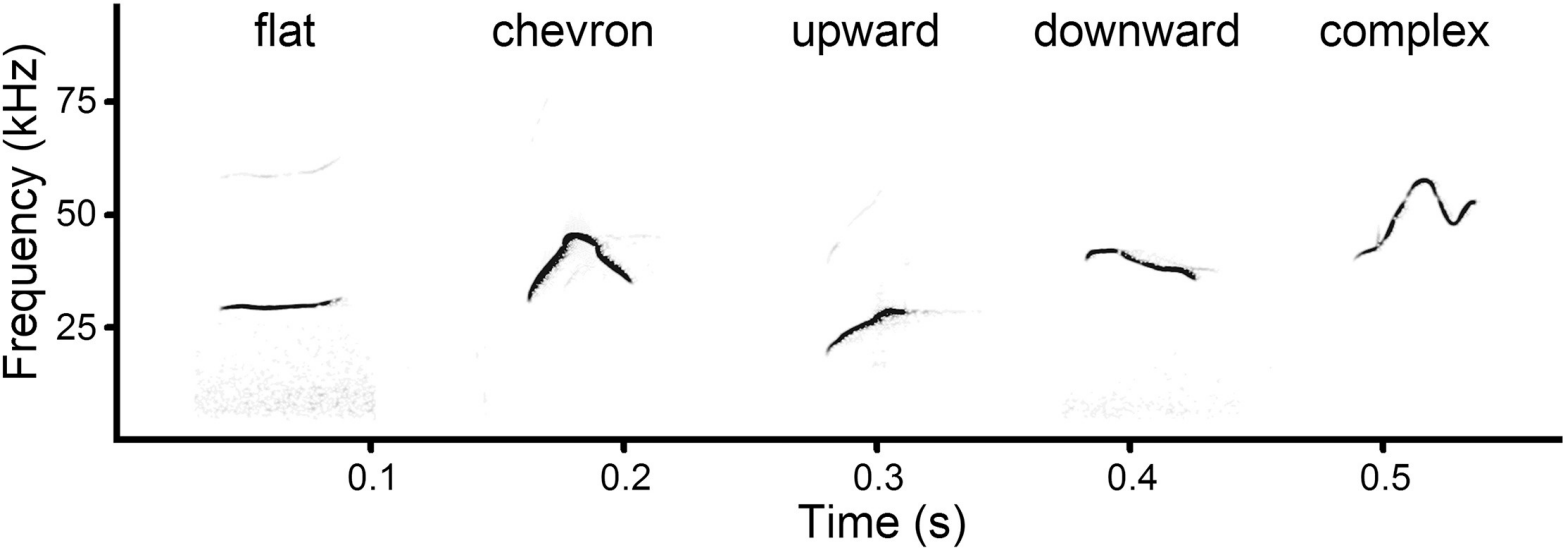
Five contour shapes occurring in USV calls of pup and adult yellow steppe lemmings: flat from 20-d pup; chevron from 4-d pup; upward from adult female; downward from 45-d pup; complex from 9-d pup. The Audio file is available at S1 Audio. Spectrogram was created using sampling frequency 192 kHz, Hamming window, FFT 1024 points, frame 50% and overlap 93.75%.

In the spectrogram window of Avisoft, we classified USV calls manually accordingly to the five f0 contour shapes: upward, flat, chevron, complex, downward (Fig 2 and S1 Audio). This classification was based (with modifications) on classifications developed for domestic mice by [7,54,61] and fat-tailed gerbils by [39]. The flat contour was denoted when the difference between f0min and f0max was less than 6 kHz. When the difference between f0min and f0max exceeded 6 kHz, the denoted contours could be the chevron (up-down one time), downward (descending from start to end), upward (ascending from start to end) or complex (up-down many times or U-shaped).

**Fig 3:**
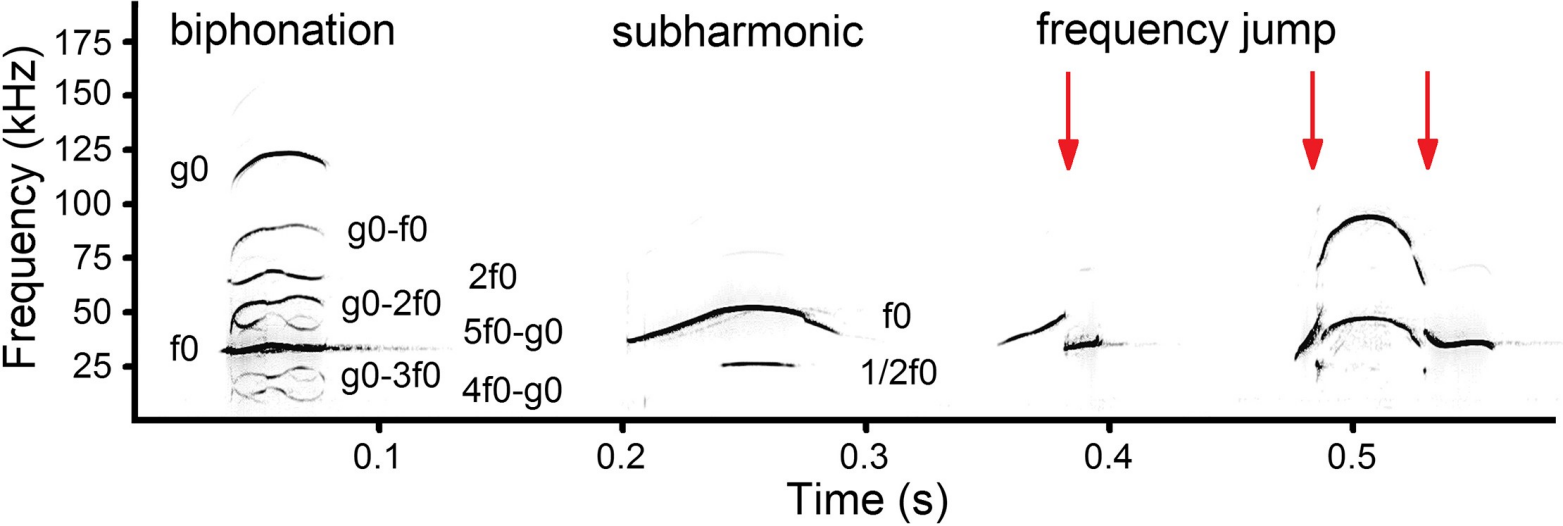
Nonlinear phenomena occurring in USV calls of pup and adult yellow steppe lemmings: biphonation from 2-d pup; subharmonic from 5-d pup; frequency jump down from 36-d pup; frequency jump down-up from 5-d pup. Designations: f0 –the low fundamental frequency band; g0 –the high fundamental frequency band; 2f0 –harmonic of f0; g0-f0, g0-2f0, g0-3f0, 4f0-g0, 5f0-g0—combinatory frequency bands; 1/2f0 –subharmonic. Red arrows indicate points of frequency jumps. The Audio file is available at S2 Audio. Spectrogram was created using sampling frequency 384 kHz, Hamming window, FFT 1024 points, frame 50% and overlap 87.5%.

For each USV call, we noted the presence of nonlinear vocal phenomena (Fig 3 and S2 Audio): frequency jumps, biphonations and subharmonics [87–89]. Frequency jump was denoted when f0 suddenly changed for ≥10 kHz up or down [7,39,54,61]. Biphonation was denoted when two independent fundamental frequencies, the low (f0) and the high (g0) and their combinatory frequency bands (g0-f0; g0-2f0; etc.) were found in a USV call [7] (Fig 3). Subharmonics were denoted when the intermediate frequency bands of 1/2 or 1/3 of f0 were found between harmonic (Fig 3).

**Fig 4:**
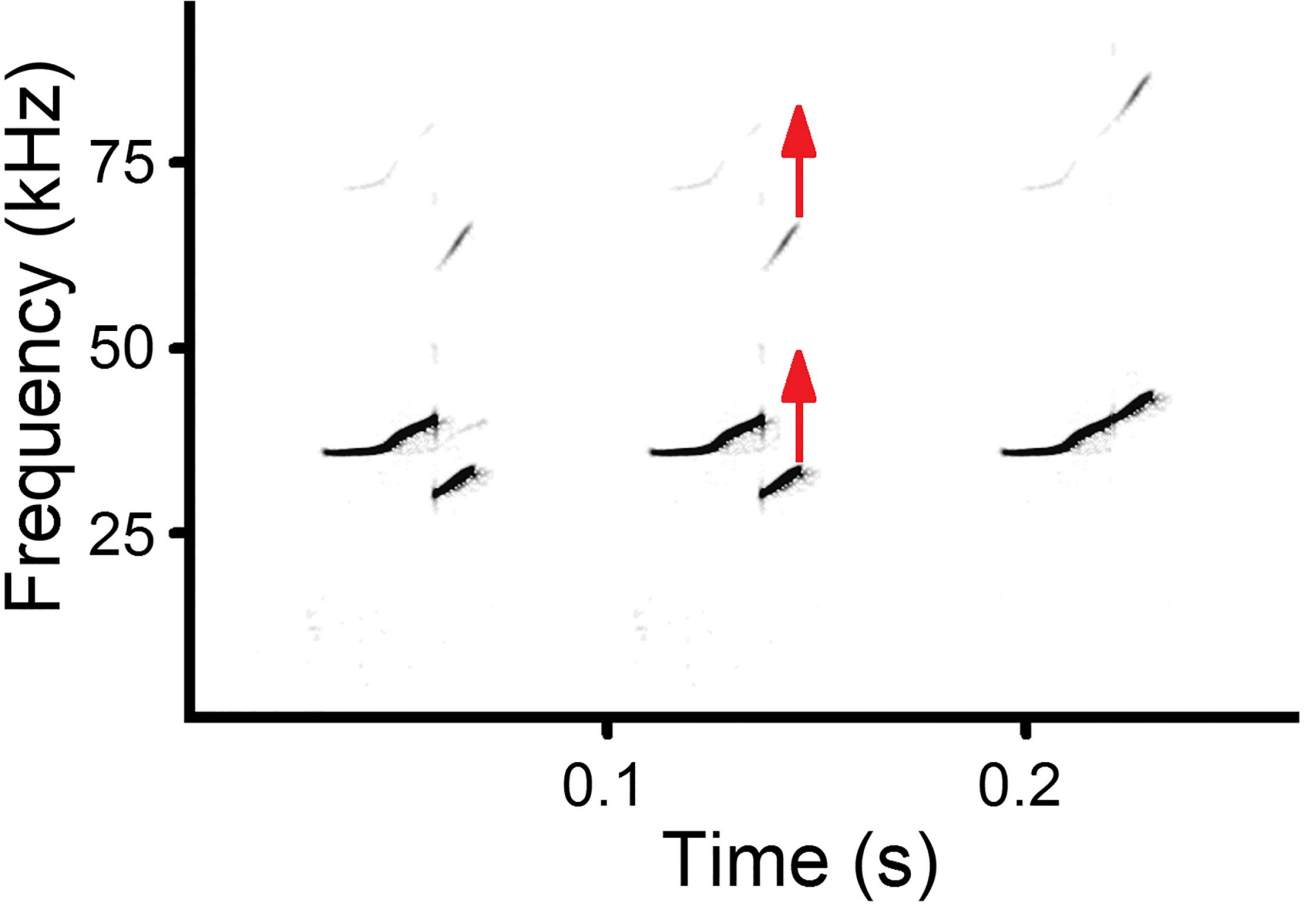
Virtual smoothing for identifying contour shape in USV calls with frequency jump. Left: the actual contour with frequency jump down from 25-d pup; Middle and right: virtual smoothing for identifying the upward contour shape. Red arrows indicate direction of smoothing for the fundamental frequency and first harmonic. Spectrogram was created using sampling frequency 192 kHz, Hamming window, FFT 1024 points, frame 50% and overlap 93.75%.

For calls with frequency jumps, we identified the contour shape by virtual smoothing the contour as if frequency jump was lacking and the fundamental frequency contour was continuous (Fig 4). The biphonic calls with two different fundamental frequency contours were classified based on the lowest frequency contour. In the biphonic calls where the high fundamental frequency (g0) contour was well visible, we additionally measured the maximum high fundamental frequency (g0max) with the reticule cursor in the spectrogram window of Avisoft.

### Statistical analyses

Statistical analyses were made with STATISTICA, v. 8.0 (StatSoft, Tulsa, OK, USA), all means are given as Mean ± SD. Significance levels were set at 0.05, and two-tailed probability values are reported. For each subject individual, the averaged values of each acoustic variable over 10 calls were used for the statistic comparisons, to decrease the number of degrees of freedom for more robust results.

We used one-way ANOVA with Tukey HSD (Honestly Significant Difference) test to estimate the effects of sex and age on the variables of body size and on the acoustics of the USV calls. We used one-way ANOVA to compare the f0 acoustics between the biphonic and non-biphonic USV calls in 1–4 d pups. We used Principal Component Analysis (PCA) to estimate the degrees of correlation between the five body size variables and for calculating the body size index on the basis of these variables. We used Pearson correlation with Bonferroni correction to estimate potential correlation between age, body size index and the acoustics of the USV calls.

## Results

### Body variables

Litter size varied from 1 to 6 pups, 3.18 ± 1.33 pups on average. The effect of particular parental pair on litter size lacked (*F*_9,45_ = 1.03, *p* = 0.43). The eyes opened from 9 to 12 d of age, at 12 d of age the eyes were opened in all pups.

ANOVA showed that pup sex (at 25–60 d, when sex could already be determined reliably) did not influence body weight (*F*_1,47_ = 0.16, *p* = 0.69), body length (*F*_1,47_ = 0.76, *p* = 0.39), head length (*F*_1,47_ = 0.02, *p* = 0.88), foot length (*F*_1,47_ = 0.19, *p* = 0.67) and tail length (*F*_1,47_ = 0.12, *p* = 0.73). Similarly, adult sex did not influence body weight (*F*_1,8_ = 1.88, *p* = 0.21), body length (*F*_1,8_ = 3.80, *p* = 0.09), head length (*F*_1,8_ = 0.63, *p* = 0.45), foot length (*F*_1,8_ = 0.18, *p* = 0.68) and tail length (*F*_1,8_ = 2.27, *p* = 0.17). Therefore, we pooled data from both sexes for further analyses.

We found a significant effect of age class on body weight and body size variables in yellow steppe lemmings (Table 1). From birth to 40 d of age, body weight gain was 1 g per day on average. In adults, significantly higher values than that of the younger age classes were observed for body weight (Table 1, *p* < 0.001, Tukey *post hoc*) and for all body variables (Table 1, *p* < 0.05, Tukey *post hoc*), for the exclusion of foot length (Table 1, *p* = 0.23, Tukey *post hoc*). We found a positive correlation between age class and body weight (*r* = 0.837, *p* < 0.001), body length (*r* = 0.930, *p* < 0.001), head length (*r* = 0.888, *p* < 0.001), foot length (*r* = 0.872, *p* < 0.001) and tail length (*r* = 0.892, *p* < 0.001). Therefore, body weight and all body variables provided clear correlates of animal age.

**Table 1:** Values (Mean±SD) of body weight and body size variables of yellow steppe lemmings at 12 age classes and one-way ANOVA results for the effect of age class on their values.

Age class (days)	*n*	Body weight (g)	Body length (mm)	Head length (mm)	Foot length (mm)	Tail length (mm)
1–4	10	6.0 ± 1.1	43.0± 4.6	16.5±2.0	8.4±0.9	4.7±0.7
5–8	10	11.8±3.6	57.5±7.0	21.9±2.8	12.6±1.8	7.7±1.8
9–12	10	14.3±2.9	69.5±5.0	23.0 ±1.7	14.2±0.8	10.3±1.4
13–16	10	19.7±4.4	76.0±5.4	26.9±1.4	16.9±1.4	13.5±1.5
17–20	10	20.5±6.5	77.9±6.8	27.7±2.1	17.5±1.2	13.7±2.0
21–24	10	30.0±10.8	84.1±7.4	28.3±1.6	18.5± 1.5	14.6 ±2.2
25–28	10	31.8±5.2	89.2±3.4	28.9±1.8	18.9±1.3	15.0±09
29–32	10	38.4±3.9	91.1±4.6	30.1±1.3	19.6±0.8	17.0±2.0
33–36	10	37.0±6.4	97.2±7.2	29.2 ±2.1	20.0±1.1	16.2 ±1.9
37–40	10	45.9±5.6	100.4±4.1	32.4±1.9	19.9±0.7	18.1±2.2
41–60	10	48.6±3.8	104.6 ±4.3	32.8±1.5	20.4 ±1.0	18.5± 2.3
Adults	10	99.0 ± 20.7	135.5± 5.8	36.6 ± 3.1	21.9± 1.5	21.5± 2.4
ANOVA		*F*_11,108_ = 96.2, *p*<0.001	*F*_11,108_ = 180.0, *p*<0.001	*F*_11,108_ = 71.2, *p*<0.001	*F*_11,108_ = 102.3, *p*< 0.001	*F*_11,108_ = 65.3, *p*<0.001

Designations: *n* = number of individuals.

Body weight and all other body variables were correlated with the first PCA axis very highly, with correlation coefficients from 0.90 to 0.98. As soon as the first PCA axis responded for 90.2% of variation, we used it as a generalizing body size index in the statistical analyses.

### Categories of USV calls

In the total sample of 1176 USV calls of all 120 subjects in the 12 age classes, the most widespread was the upward contour: 721 USV calls (61%), then in order flat contour: 251 USV calls (21%), chevron: 134 USV calls (11%), complex: 45 USV calls (4%) and downward: 25 USV calls (2%).

**Fig 5:**
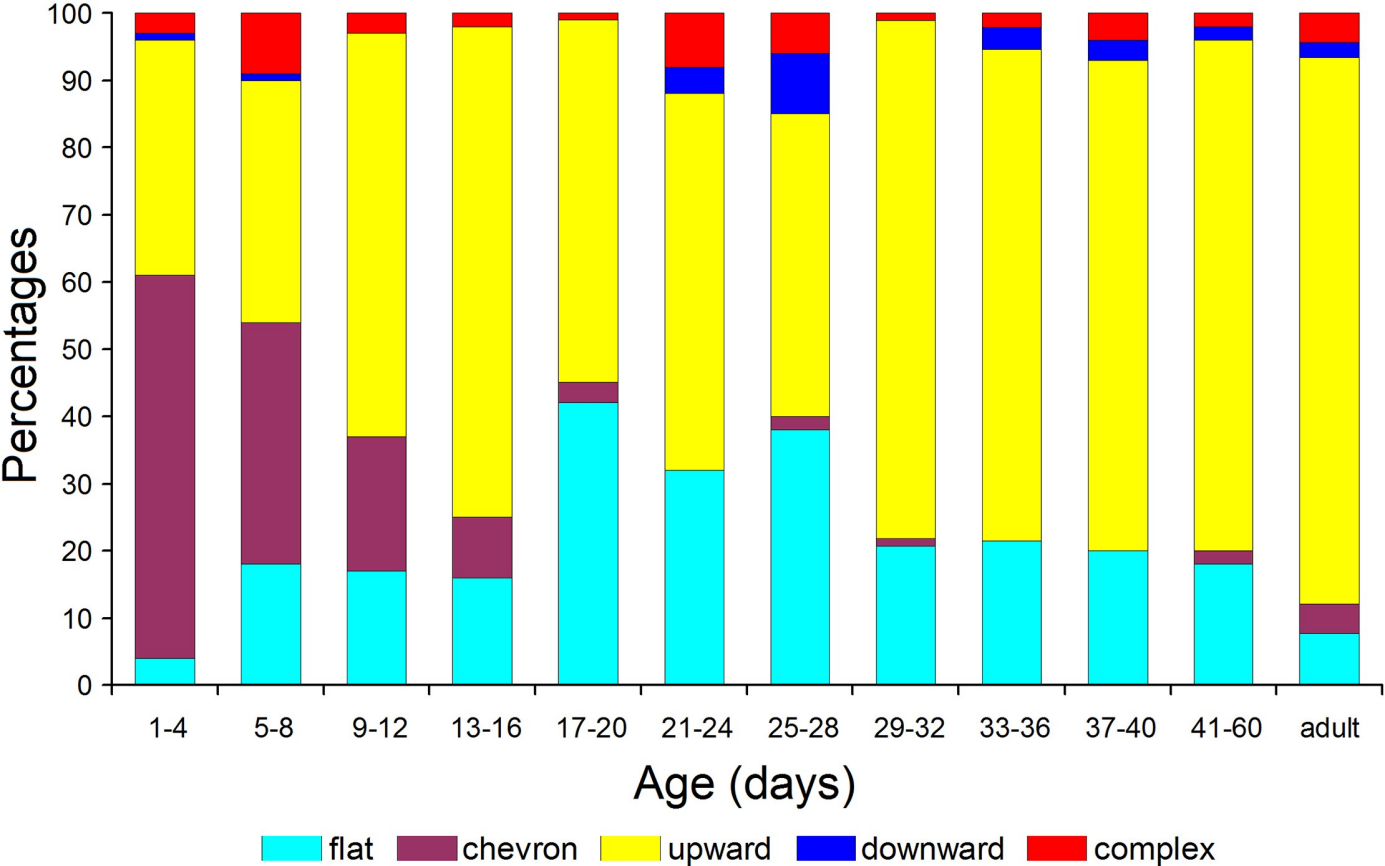
Percentages of five different USV contour shapes in the total sample of 1176 USV calls from the 120 subject yellow steppe lemmings at 12 age classes.

Pups at 1–4 d were distinctive by prevalence of the chevron USV contour (57%), whereas at 5–8 d, the chevron and upward contours were equally frequent (35–36%) (Fig 5). At older age classes, the upward contour prevails (from 45 to 76%), and in adults it was found in 74% of USV calls (Fig 5). Flat contour was least frequent (4%) at 1–4 d age class but was second most common after the upward contour at the older age classes (Fig 5).

Nonlinear phenomena occurred at all age classes, in 389 (33%) USV calls from the total of 1176 USV calls. Most frequent were frequency jumps: 372 (32%) USV calls, whereas biphonations were presented in 43 (4%) and subharmonics in 13 (1%) USV calls. Thirty nine (3.3%) of USV calls contained two nonlinear phenomena, frequency jump and biphonation.

**Fig 6:**
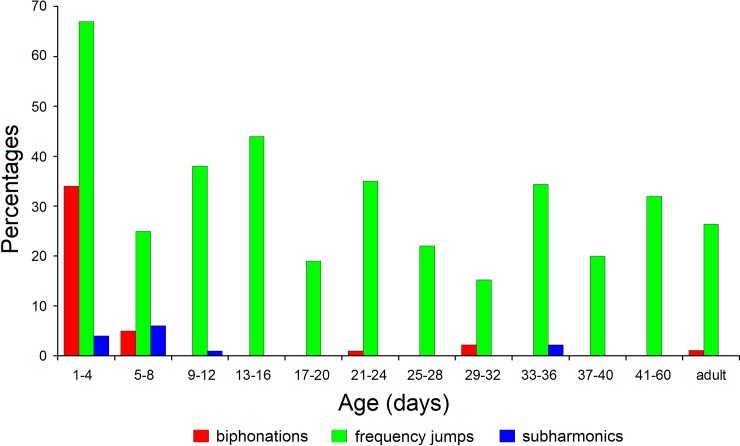
The occurrence of nonlinear phenomena by age classes in the total sample of 1176 USV calls from the 120 subject yellow steppe lemmings at 12 age classes. For each age class, the percent sum is not equal to 100%, as 39 USV calls contained two nonlinear phenomena.

Pups at 1–4 d were distinctive among other age classes with the highest percentage (76%) of USV calls with different nonlinear phenomena and in particular with the highest percentage (34%) of USV calls with biphonations (Fig 6). Among other age classes, biphonations and subharmonics were presented in 5-8-d pups, lacking practically at older ages. Since 9–12 d of age onwards, the USV calls of yellow steppe lemmings contained nearly exclusively frequency jumps; percentages of calls with frequency jumps ranged from 15% to 44% depending on age class. In USV calls of adults, amount of nonlinear phenomena comprised 28% (Fig 6). Therefore, USV calls of the youngest age classes (1–4 d and 5–8 d) were distinctive from those of the older ages.

### Acoustic variables

**Fig 7:**
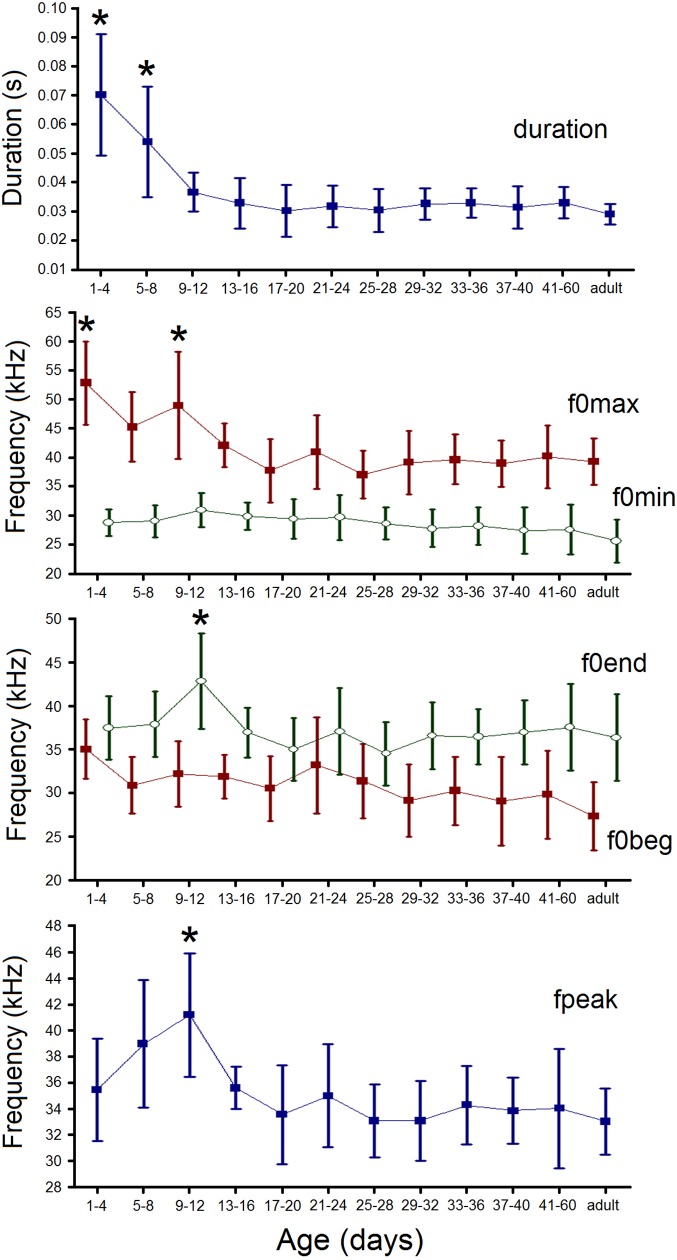
Changes in values of acoustic variables of yellow steppe lemming USV calls across age classes. Designations: duration–call duration; f0max–the maximum fundamental frequency; f0min–the minimum fundamental frequency; f0beg–the fundamental frequency at the onset of a call; f0end–the fundamental frequency at the end of a call; fpeak–the frequency of maximum amplitude; central points–means, whiskers–SD. Asterisks indicate the age classes, which are significantly different from other age classes by the given acoustic variable (*p* < 0.05, Tukey *post hoc*). No asterisks indicate the age classes, which do not differ from adults and from each other.

Age class significantly affected all acoustic variables for the exclusion of the f0min, for which the effect of age class was marginally significant (Table 2). The duration of USV calls significantly decreased from the age class of 1–4 d to the age class of 9–12 d. Since the age 9–12 d, pup USV duration was becoming undistinguishable from those in adult USV calls (Fig 7). The maximum fundamental frequency of USV calls also decreased with age, displaying significantly highest values at 1–4 d of age and at 9–12 d of age and showing undistinguishable values between pups and adults since 13–16 d of age onwards (Fig 7). The values of the minimum and of the start fundamental frequencies did not display significant changes with age (Fig 7). The values of the end fundamental frequency and of the peak frequency did not show significant changes with age as well, for exclusion of an elevation at 9–12 d of age, during which they differed significantly from the values of pups from both the younger and older age classes and from adults (Fig 7). The elevation of the end fundamental frequency and of the peak frequency from the age of 1–4 d to the age of 9–12 d could result from the shift by pups from prevalent using USV with chevron contour (with frequency humps down) to prevalent using USV with upward contour (Figs 5 and 6).

**Table 2:** Values (Mean±SD) of USV acoustic variables of yellow steppe lemmings at 12 age classes and one-way ANOVA results for the effect of age class on their values.

Age class (days)	*n*	Duration (s)	f0beg (kHz)	f0max (kHz)	f0end (kHz)	f0min (kHz)	fpeak (kHz)
1–4	10	0.070±0.021	35.1±3.4	52.9±7.2	37.5±3.7	28.9±2.3	35.5±3.9
5–8	10	0.054±0.019	30.9±3.2	45.3±6.0	37.9±3.7	29.1±2.8	39.0±4.9
9–12	10	0.037±0.007	32.2±3.8	49.1±9.3	42.9±5.5	31.1±2.9	41.2±4.7
13–16	10	0.033±0.009	31.9±2.5	42.2±3.8	37.0±2.8	29.9±2.3	35.6±1.6
17–20	10	0.030±0.009	30.5±3.7	37.8±5.5	35.0±3.6	29.5±3.4	33.6±3.8
21–24	10	0.032±0.007	33.2±5.5	41.0±6.4	37.1±5.0	29.7±3.9	35.0±3.9
25–28	10	0.030±0.007	31.4±4.3	37.1±4.1	34.5±3.6	28.7±2.8	33.1±2.8
29–32	10	0.033±0.006	29.2±4.2	39.2±5.5	36.6±3.9	27.9±3.2	33.1±3.0
33–36	10	0.033±0.005	30.2±3.9	39.7±4.3	36.5±3.2	28.3±3.2	34.3±3.0
37–40	10	0.031±0.007	29.1±5.1	39.0±4.0	37.0±3.7	27.5±4.0	33.9±2.5
41–60	10	0.033±0.005	29.8±5.1	40.2±5.4	37.6±5.0	27.7±4.3	34.0±4.6
Adults	10	0.029±0.004	27.3±3.9	39.4±4.0	36.4±5.0	25.7±3.7	33.0±2.5
ANOVA		*F*_11,108_ = 14.4, *p*<0.001	*F*_11,108_ = 2.84, *p* = 0.008	*F*_11,108_ = 7.16, *p*<0.001	*F*_11,108_ = 2.48, *p* = 0.008	*F*_11,108_ = 1.80, *p* = 0.06	*F*_11,108_ = 5.01, *p*<0.001

Designations: *n*–number of individuals; duration–call duration; f0beg–the fundamental frequency at the onset of a call; f0end–the fundamental frequency at the end of a call; f0max–the maximum fundamental frequency; f0min–the minimum fundamental frequency; fpeak–the frequency of maximum amplitude.

For the age class of 1–4 d, we could measure the values of the maximum high fundamental frequency (g0max) for 33 of the 34 biphonic calls. For other age classes, the g0max could only be measured in two of the nine biphonic calls, so we omitted these insufficient data from analyses. At the 1–4 d age class, the biphonic calls were presented in 9 of the 10 study pups. The mean g0max of the biphonic calls was 121.7±8.8 kHz. Comparison of acoustic variables between the biphonic and non-biphonic USV calls of the 1–4 d pups showed that duration, f0beg, f0max and f0end did not differ between the biphonic and non-biphonic USV calls (Table 3). However, the f0min and fpeak were lower in the biphonic calls than in non-biphonic USV calls (Table 3).

**Table 3:** Values (Mean±SD) of USV acoustic variables of yellow steppe lemmings at biphonic and non-biphonic USV calls of 1–4 d pups and one-way ANOVA results for their comparison.

USV calls	*n*	Duration (s)	f0beg (kHz)	f0max (kHz)	f0end (kHz)	f0min (kHz)	fpeak (kHz)
biphonic	34	0.076±0.023	35.1±4.5	54.8±10.5	36.1±5.7	27.3±2.5	32.4±4.1
non-biphonic	66	0.067±0.029	35.1±4.5	51.9±8.9	38.2±7.8	29.6±4.4	37.0±7.2
ANOVA		*F*_1,98_ = 2.87, *p =* 0.09	*F*_1,98_ = 0, *p* = 1	*F*_1,98_ = 2.12, *p* = 0.15	*F*_1,98_ = 1.87, *p* = 0.17	*F*_1,98_ = 7.93, *p* = 0.006	*F*_1,98_ = 12.23, *p*<0.001

Designations: *n*–number of USV calls; duration–call duration; f0beg–the fundamental frequency at the onset of a call; f0end–the fundamental frequency at the end of a call; f0max–the maximum fundamental frequency; f0min–the minimum fundamental frequency; fpeak–the frequency of maximum amplitude.

Both age class and body size index significantly negatively correlated with all acoustic variables of USV calls, for the exclusion of f0end (Table 4). Therefore, the values of duration, peak frequency and of most variables of fundamental frequency of yellow steppe lemming USV calls decreased with increasing age and body size.

**Table 4:** Pearson’s correlation coefficients between age class, body size index and USV acoustic variables.

Parameter	*n*	Duration	f0beg	f0max	f0end	f0min	fpeak
Age class	120	*r* = -0.53, *p*<0.001	*r* = -0.36, *p*<0.001	*r* = -0.47, *p*<0.001	*r* = -0.16, *p* = 0.088	*r* = -0.31, *p*<0.001	*r* = -0.38, *p*<0.001
Body size index	120	*r* = -0.64, *p*<0.001	*r* = -0.42, *p*<0.001	*r* = -0.53, *p*<0.001	*r* = -0.17, *p* = 0.061	*r* = -0.31, *p*<0.001	*r* = -0.37, *p*<0.001

Threshold for significant values after Bonferroni correction comprises *p* < 0.008. Designations: *n*–number of individuals; duration–call duration; f0beg–the fundamental frequency at the onset of a call; f0end–the fundamental frequency at the end of a call; f0max–the maximum fundamental frequency; f0min–the minimum fundamental frequency; fpeak–the frequency of maximum amplitude.

## Discussion

This study provides categorization and spectrographic analyses of USV calls of yellow steppe lemmings from neonates to breeding adults. The isolation-induced USV calls emerged since 1st day of pup life and occurred at all age classes and in both sexes. Rapid physical growth and the early (9–12 d) eyes opening correlated with the early (9–12 d) emergence of mature vocal patterns of USV calls. The mature vocal patterns included a prominent shift from chevron to upward contours, almost complete disappearance of biphonation and the shortening of duration and decrease of fundamental frequency of USV calls. In addition, this is the first study describing the acoustic variables of the biphonic USV calls in a rodent species.

### Rapid physical growth and early eyes opening

Our data regarding the rapid physical growth (1 g body weight gain per day from birth to 40 d of age) are consistent with earlier data reporting the similarly fast postnatal body weight gain in captive pup yellow steppe lemmings originated from the same natural population [78]. Among 39 investigated Arvicolinae species, the absolute body weight gain is faster (1.14 g per day) only in the European water vole *Arvicola amphibious* [90–92].

In the relatively large-sized yellow steppe lemming (99 g), the eyes opened early, at 9–12 d of age. This occurs approximately at the same age as in the *Scotinomys teguina* singing mice (10–13 d) and earlier than in the *S*. *xerampelinus* singing mice (19 d), which both are much smaller (about 10–15 g) [49]. In domestic mice, weighting about 20 g, the eyes open between 10 and 16 d of age [7]. In pup fat-tailed gerbils, the rodent of comparable size (60–81 g) with the yellow steppe lemming (100–120 g), the eyes open between 16 and 24 d of age [93,94].

### Ontogeny towards shorter and low-frequency USV

In yellow steppe lemmings, age and body size index significantly negatively correlated with all acoustic variables of USV calls for the exclusion of the end fundamental frequency. Pup USV calls were longer and higher-frequency than in adults. The observed trajectories of ontogenetic changes towards the shorter and lower-frequency USV calls were similar to those reported for the audible squeaks of the yellow steppe lemmings [80]. Ontogenetic changes of USV calls in yellow steppe lemmings were overall similar with those in laboratory domestic mice. In mice, the USV calls also shorten and decrease in frequency with age, although each call type can display a specific pattern of developmental changes [7,23,25,26,28–30,32,33]. In domestic mice, the developmental analyses are complicated, because the ontogenetic trends of USV acoustic variables are strain-specific, although generally follow the species-specific pattern [31,37,95].

In other rodents, different ontogenetic pathways of USV development from pups to adults are reported. A pathway towards the shorter and higher-frequency USV calls was observed in a Gerbillinae rodent, the fat-tailed gerbil [39]. In 1–10 d fat-tailed gerbils, the USV calls were longer (50.0 ms) and lower-frequency (52.2 kHz) than in the adults (22.0 ms and 66.8 kHz respectively) [39]. Another different ontogenetic pathway (towards the longer and lower-frequency USV calls) is characteristic of laboratory rats [1,12,47,48,50,51,53,55,56,58,96–98]. Rat pup 40-kHz USV calls decrease in frequency during the first 2–3 wk of life [12,47,50] followed by increase in frequency at about 4 wk [12,48,53], and splitting after 4 wk to mature vocal patterns of 22-kHz and 50-kHz USV calls [1,51,58,96,97], which both display a further decrease of fundamental frequency to senescence [55,56,98]. At the same time, duration of rat USV calls remains stable until 3 wk, then suddenly decreases [12] and increases again from 6 wk up to senescence [55,56,98]. In ontogeny of *Peromyscus californicus* rodents, USV calls shorten since 2–4 d (150 ms) to 28 d (about 20 ms), whereas the fundamental frequency changes inconsistently, first increasing from 2–4 d to 7 d of age and then decreasing back to the same values to 21–28 of age [57].

### Re-structure of communication with eyes opening

This study revealed that in yellow steppe lemmings, the age of eyes opening (9–12 d) coincides with an abrupt transition from juvenile to mature vocal patterns of USV calls. As soon as in newborn pups the primary function of the isolation USV calls is eliciting pup retrieval by parents at falling out of the nest or in another critical for survival situation [99,100], we can propose that eyes opening and the adding of the visual communicative channel declines the importance of the auditory channel in mother-offspring communication. Eyes opening in rodents occurs very fast, over a period of one to two days [101] and drives multiple neuronal changes [102] influencing ability to orientation [103]. Indirect support for this proposal comes from decline of emission of isolation-induced USV calls after eyes opening in common voles *Microtus arvalis* [28], mandarin voles *Lasiopodomys mandarinus* [104], fat-tailed gerbils [105], Mongolian gerbils *Meriones unguiculatus* [28,106], Syrian hamsters [28], domestic mice [7,28], and laboratory rats [28]. Pups after eyes opening are more mobile and can return to the nest ourselves; their thermoregulatory ability is substantially better than in newborns [107,108]. Pups become less dependent on parents, and their USV calls respectively loss the infantile traits. Potentially, dark-rearing experiments can reveal the effects of visual stimuli with eye opening on maturation of USV characteristics in yellow steppe lemmings and other laboratory rodents.

### Transit from infantile to mature USV

In yellow steppe lemmings, the abrupt transition from juvenile to mature patterns of USV calls occurred over a short period of a few days, from 9 to 12 d of age, after that they become undistinguishable from USV calls of adults. The fast transition from infantile to mature USV patterns was well detectable based on prevalent USV contours, amount of the nonlinear phenomenon biphonation and on USV acoustics.

The transition to the mature vocal patterns of USV calls involves the restructuring of the isolation-induced USV calls turned from the “pup” type (with prevailing the chevron contour) to the “adult” type (with prevailing the upward contour). Ontogenetic changes in percentages of different contours were also reported in domestic mice [54], Norway rats [58,65] and fat-tailed gerbils [39]. In fat-tailed gerbils, the contours flat and chevron were also more frequent in pups than in adults whereas the contours upward, short and complex were more frequent in adults than in pups [39].

Transition to the mature vocal patterns of USV calls in yellow steppe lemmings was accompanied by almost complete disappearance of the nonlinear phenomenon biphonation. Biphonation is recognizable by presence in call spectrum of two independent fundamental frequencies, which interact to each other with creation of combinatory frequency bands, resulting in strong complication of call structure [88,109,110]. Biphonations occur rarely in USV calls of wild-type rodent pups (e.g., in pup fat-tailed gerbils only in 2 of 782 USV calls [39]), but are often in pup laboratory mice belonging to strains with autism [7]. In pup yellow steppe lemmings, the relatively high percentage of USV calls with biphonations at 1–8 d of age can be due to both imperfect control on vocal production and the acting of mechanism for decreasing vocal monotony for preventing habituation and enhancing attention of parents to a pup in a critical for it situation [88]. The values of acoustic variables did not differ between the biphonic and non-biphonic USV calls of 1–4 d pups, for the exclusion of the lower f0min and f0peak in the biphonic calls.

In addition, the transition to mature vocal patterns of USV calls in yellow steppe lemmings is related to decreasing the duration and maximum fundamental frequency to values characteristic of adults. These changes in USV acoustics occur within a short time span from birth to 12 d of age, and after completing this period, the physical growth of pups continues with the same speed although the acoustics of USV calls remain unchanged. We can therefore conclude that the speed of development of mature patterns of USV calls significantly exceeds the speed of physical growth in the yellow steppe lemming. Consistently, in *Scotinomys* singing mice, the vocal traits of adult songs emerge in pups earlier than they complete their physical growth [49]. Potentially, the transit from infantile to mature vocal patterns in rodent USV calls is due to the developmental changes of the larynx, as was convincingly demonstrated for the audible contact calls of common marmosets *Callitrix jaccus* [111] and goitred gazelles *Gazella subgutturosa* [112].

### Yellow steppe lemming model of USV ontogeny

The isolation-induced USV calls of yellow steppe lemmings emerged since 1^st^ day of pup life. In other rodents (gerbils, mice, California mice, voles, rats, hamsters), the isolation-induced USV calls emerge since 1^st^-3^th^ day of life, depending on the species [26,46,104,106,113–124], for exclusion of fat-tailed gerbils, in which the isolation-induced USV calls emerge only since 5^th^ day of life [39].

The isolation-induced USV calls of yellow steppe lemmings occurred at high rates at all ages. The unified 2-min isolation procedure on an unfamiliar territory was equally applicable for inducing USV calls across age classes from newborns to adults. This makes the yellow steppe lemming a very convenient cross-age and cross-sex animal model of USV ontogeny. At the same time, for the mice model, the isolation procedure is inapplicable for all ages because of a low USV call rate at older ages [10,125]. Although the isolation-induced USV calls reported not only for pup but also for adult mice [5,125], for most individual adolescent and adult mice the isolation or restraint procedure is ineffective for inducing the USV calls and some kind of social stimulation from conspecifics is necessary to provoke the ultrasonic vocalization [10,37,125–130].

The isolation-induced USV calls of yellow steppe lemmings occurred across ages in both sexes. In contrast, mice model of USV ontogeny is mostly limited with male sex [95,131], as female mice produce USV calls at rates much lower than male mice [86,95,127,128,131–136] in spite of the structural similarity of USV calls between sexes in mice [135].

Adult *Glaucomys* flying squirrels and adult *Typhlomys* dormice can also produce USV calls during isolation procedure in the lab [70,137,138]. However, for some other rodent species, the isolation procedure is ineffective for inducing the USV calls in adults, as e.g. for Mongolian gerbils [84,139], fat-tailed gerbils [39] and for North African gerbils *Dipodillus campestris* (unpublished data of the authors), whereas in pups of these species the isolation-induced USV calls are usual [28,39,105,140].

We applied in this study the cross-sectional approach, with each individual tested ones at one of 12 age classes, covering ontogeny from neonates to adults. The alternative longitudinal approach, using the same individuals repeatedly tested in different ages, allows tracking the ontogenetic changes at individual level [39,131]. However, the longitudinal approach does not avoid potential effects of habituation to test procedure on vocal and physical development of study animals [125,130,141,142]. The use of the cross-sectional approach with many non-overlapping age classes (age-class slicing) allows avoiding the potential effects of habituation to test procedure and at the same time enables to track the ontogenetic changes along the entire development, thus combining the advantages of both the cross-sectional and longitudinal approaches. However, for such studies are only appropriate the species breeding at high rate in captivity, as the yellow steppe lemming.

## Supporting information

S1 Table Mean values for acoustic variables of USV calls, body weight, body length, head length, foot length, tail length and body size index for 120 individual pup and adult yellow steppe lemmings. (XLS)

S1 Audio USV calls of yellow steppe lemmings exemplifying the five contour shapes. USV with contour flat from 20-d pup; USV with contour chevron from 4-d pup; USV with contour upward from adult female; USV with contour downward from 45-d pup; USV with contour complex from 9-d pup. Order as on Fig 2. Sampling frequency of the acoustic file is 192 kHz.
(WAV)

S2 Audio USV calls of yellow steppe lemmings exemplifying the three kinds of nonlinear phenomena. USV call with biphonation from 2-d pup; USV call with subharmonic from 5-d pup; USV call with frequency jump down from 36-d pup; USV call with frequency jump down-up from 5-d pup. Order as on Fig 3. Sampling frequency of the acoustic file is 384 kHz.
(WAV)
